# Quantitative determination of leptin hormone using gold nanoparticle-based lateral flow assay

**DOI:** 10.1007/s00604-024-06945-6

**Published:** 2025-01-09

**Authors:** Erhan Zor, Sabri Alpaydin, Haluk Bingol

**Affiliations:** 1 Science and Technology Research and Application Center (BITAM), Necmettin Erbakan University, Konya, Türkiye; 2 Department of Science Education, A.K. Education Faculty, Necmettin Erbakan University, Konya, Türkiye; 3 Department of Chemistry Education, A.K. Education Faculty, Necmettin Erbakan University, Konya, Türkiye; 4 Department of Basic Sciences, Faculty of Engineering, Necmettin Erbakan University, Konya, Türkiye

**Keywords:** Lateral flow assay, Colorimetric lateral flow reader, Leptin hormone, Gold nanoparticle

## Abstract

A lateral flow assay (LFA) has been developed that can be used in point-of-care (PoC) use for the sensitive determination of leptin hormone. The limit of detection value was 0.158 ng/mL and the limit of quantification value was 0.479 ng/mL for leptin hormone. The stability studies showed that the assay response was equal to the initial value even after 6 months. LFA accuracy tests performed in commercial artificial serum samples were compared with the enzyme-linked immunosorbent assay (ELISA). The obtained recovery values between 95 and 110% show that the developed LFA can be used for quantitative determination of leptin hormone. As the result of the study, a fast and disposable assay was developed for the determination of leptin hormone, which can be performed with a small amount of sample in less than 15 min without the need for long-term analysis stages, qualified personnel, expensive equipment and devices.

**Graphical Abstract:**

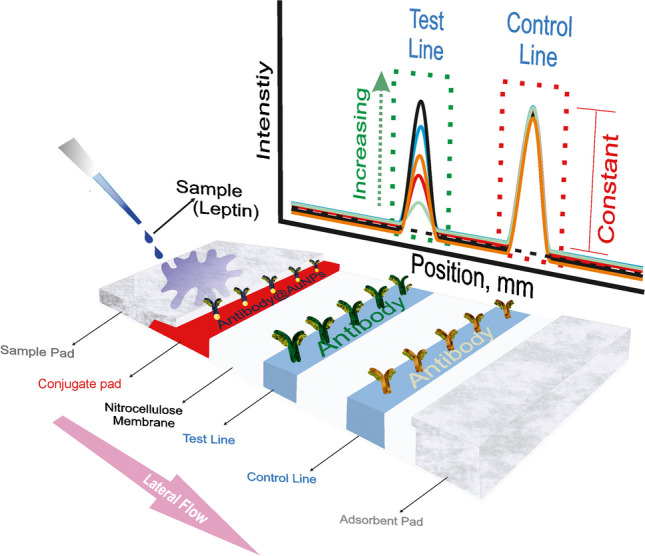

**Supplementary Information:**

The online version contains supplementary material available at 10.1007/s00604-024-06945-6.

## Introduction

The early diagnosis of diseases is a well-known critical fact. Nowadays, the majority of systems used in disease diagnosis and monitoring require expensive equipment and trained personnel, limiting people's access to these systems, especially in less developed regions [1, 2]. To overcome this problem and enable more people to benefit from diagnosis systems, many researchers have focused on the development of Point-of-Care (PoC) diagnostic kits for many years, and these studies are still relevant due to the advantages they offer [3, 4]. While there are debates about the achievement of definitive results in the field of healthcare through PoC diagnostic kits, studies in this regard have generally shown that these systems provide low cost and fast response [5, 6]. Thanks to rapidly advancing technology, PoC diagnostic technologies with different features are being developed [7].

In PoC diagnostic technologies, diversity arises from different signals as well as the variety of systems that can be developed based on the used platform structure. Among these platforms, flexible paper and polymer-based systems are particularly noteworthy. On the other hand, paper-based platforms are preferred due to their ease of availability, cost-effectiveness, passive liquid transport capability, compatibility with (bio)chemicals, and the ability to be printed and coated. Utilizing these features, paper-based platforms offer the possibility of conducting analyses with existing systems in a simple production process, low cost, and portable size. The first paper-based bioassay platform (glucose test) was based on the absorption of chemicals onto paper and the colour change when sampling was performed in 1957 [8]. The technical basis of the lateral flow immunoassay was derived from the latex agglutination assay, the first of which was developed in 1956 by Plotz and Singer [9]. Then, the first homemade LFA was performed in 1976 to detect human choronic gonadotropin (hCG) in urine [10]. Lateral flow assay (LFA) systems eliminated standard procedures such as incubation and washing steps in test strips and offered the possibility of reaching lower diagnostic limits in a short time. Initially, both test strips and lateral flow systems demonstrated the advantages of ELISA that were already in use, and many analysis processes were transferred to paper-based sensors, which were more practical than ELISA technology and did not require a specialized device. Consequently, they became widely available commercially.

LFAs, which are generally application of optical-based assays, are a type of biosensor based on the antigen–antibody interaction principle [11]. LFAs are widely preferred sensor types in PoC diagnostic technology because they do not require any device for interpreting test results. Despite efforts to develop strategies for measurement using various devices (such as mobile phones, inexpensive colorimetric devices, etc.), most of the time, LFAs perform the function of a user's visual "yes/no" (or positive/negative) test. This is because the most critical part of the evaluation are the transducers used in lateral flow systems. Colorimetric labels provide simplicity and practicality to LFA. In this sense, gold nanoparticles (AuNPs) are commonly used as colorimetric labels in LFAs [12, 13].

With the modern lifestyle characterized by high-energy food consumption and reduced physical activity, various diseases have become prevalent, including insulin resistance, diabetes, hypertension, obesity, and even orthopedic problems, collectively referred to as metabolic syndrome. Therefore, the diagnosis and treatment of obesity have become highly important nowadays. Leptin is a hormone with structural similarity to cytokines, consisting of 167 amino acids and weighing 16-kDa. It is synthesized in adipose tissue and plays a crucial role in energy regulation [14]. In addition to being primarily released by adipose tissue, it is expressed in the stomach fundus, liver, skeletal muscles, placenta [15], human ovaries [16], heart [17], human mammary glands, and gastric epithelium [15, 18]. Leptin levels are directly related to adipose tissue mass, nutritional status, body mass index, and body fat percentage [19]. Its primary function is to regulate food intake [20]. By creating a sense of fullness, leptin regulates fat storage within the organism and appetite, making it an essential "satiety hormone". In recent years, numerous studies have shown that leptin has various immunological effects [21]. Additionally, it has been suggested that leptin levels may play a role in some cancer types. Particularly, leptin is identified as a marker for colorectal cancer-derived cancers [22], and in some conditions, it may have a carcinogenic role [23]. Therefore, while the determination of leptin hormone plays an important role in obesity diagnosis (or monitoring), it can also be seen as a potential biomarker in the diagnosis of certain cancer types. Besides, a recent study has showed that leptin molecule can be used as a marker for sperm motility and DNA fragmentation before and after cryopreservation [24]. Despite its importance, when examining the tests used for leptin determination, it can be seen that immunoassay techniques such as radioimmunoassay [25], ELISA [26], and immunocytochemical assay [21] were generally used for leptin determination in the late 1990s and early 2000s. Compared to the commercial methods currently used (in hospitals and medical centers), LFAs stand out as rapid, simple, cost-effective, user-friendly systems, and there is no reported LFA for determination of leptin to the best of our knowledge.

The motivation of this study is to develop an LFA that can be used in PoC applications for the sensitive determination of leptin hormone. The methodology used in the sensor design were performed in four steps; AuNPs preparation and surface functionalization with antibody, creation of LFA platform, determination of optimal experimental conditions, qualitative and quantitative leptin detection with LFA, which was compared with the ELISA method performed in commercial artificial serum samples. As the result of the study, a fast, economical, and disposable test kit was developed for the determination of leptin hormone, which can be performed with a small amount of sample in less than 15 min without the need for long-term analysis stages, the need for trained or specialized personnel, expensive equipment and devices needed in conventional methods. Besides, this is the first study to develop an LFA for successful quantitative determination of leptin hormone.

## Experimental

### Materials and equipment

The chemicals were purchased from global suppliers. Gold(III) chloride trihydrate (HAuCl_4_·3H_2_O) was obtained from Alfa Aesar, trisodium citrate, sodium chloride, sucrose, Tween-20, phosphate-buffered saline tablets (10 mM, pH 7.4), bovine serum albumin (BSA), and Leptin Human (L4146) were purchased from Sigma-Aldrich. The anti-leptin antibody (ab16227) and anti-rabbit IgG (ab6702) were obtained from Abcam. The solutions containing prepared nanomaterials and buffer solutions were stored in a dark environment at + 4 ºC in a refrigerator. Ultra-pure water (resistance 18.2 M Ω cm) obtained with a Direct-Q3 (Millipore) apparatus was freshly prepared before each experiment. The glass fiber used as the conjugate pad (GFCP00080000), the cellulose fiber used as the sample and waste (adsorbent) pad (CFSP001700), the nitrocellulose membrane used as the detection pad, and the backing card (HF090MC100) were all purchased from Millipore.

AuNPs were size-characterized using a Field Emission Scanning Electron Microscope (with FESEM-STEM detector)/ZEISS GeminiSEM 500. The spectrophotometric characterization of AuNPs was carried out using an Ultraviolet–Visible Spectrophotometer (UV–Vis) (Shimadzu UV-1800). The deposition of test and control line antibodies in a uniform and homogeneous manner was performed using a Linomat 5 (Camag). The measurement of the response of the LFA (intensity of test and control lines) was conducted using a Colorimetric Lateral Flow Reader (ESEQuant). For all the measurements, the background signal of blank (PBS) was subtracted. The drying of the sample pad and conjugate pad was accomplished in a vacuum oven. The functionalization of AuNPs with antibodies was carried out using a thermal shaker (Biosan TS-100C). The centrifuge process for antibodies conjugated-AuNPs was performed using a centrifuge machine (HETTICH). The antibodies, solutions, and prepared test kits were stored in a refrigerator (LIEBHERR).

### Setup the lateral flow assay

The LFA comprises six essential components: The sample pad, conjugate pad, detection pad, test line, control line, and adsorbent pad. To construct LFA platform, it is necessary to prepare these components. The synthesis and the surface modification of colloidal AuNPs were respectively shown in Fig. S1 and Fig. S2. The sample pads were respectively washed with distilled water, 10 mM pH 7.4 phosphate buffered saline (PBS) solution, PBS with Tween 20 (PBST; Tween-20 was optimized as 0.05%), and immunobuffer (PBST + BSA; BSA was optimized as 3%), and then dried in an incubator at 37 ºC for 1 h [12] as shown in Fig. S3. The detection pad was washed with immunobuffer solution and dried in an incubator at 37 ºC (Fig. S4a-b). After the drying process, the test and control lines were loaded smoothly and uniformly using a sample spraying device (Fig. S4c-e), and then dried in an incubator at 37 ºC for 1 h (Fig. S4f). The glass fiber to be used as the conjugate pad was washed with immunobuffer solution and dried at 37 ºC (Fig. S5a-b). After the drying process, the glass fiber was immersed in the Ab@AuNPs solution (Fig. S5c-d). The glass fiber was dried under vacuum at 37 ºC for 1 h (Fig. S5e-f).

In the process of creating the LFA platform, as described above, the washed and dried sample pad, adsorbent pad, and conjugate pad were assembled on the detection pad. Initially, the adhesive tapes on the detection pad were peeled, and the conjugate pad was placed. Then, a portion of the adsorbent pad was attached to the membrane, aligning with the conjugate pad, and excess material was trimmed. The adhesive tape on the bottom side of the detection pad was peeled, the sample pad was attached to the bottom of the membrane, and excess material was trimmed. The entire platform created on the detection pad was cut using a guillotine. LFA was cut to 6 mm of thickness for qualitative and quantitative tests. The conducted studies resulted in well-drawn and homogeneous test and control lines, and the conjugate pad was prepared using approximately 1.6 mL of functionalized AuNPs for one complete membrane.

### Working principle of LFA for leptin detection

**Fig. 1:**
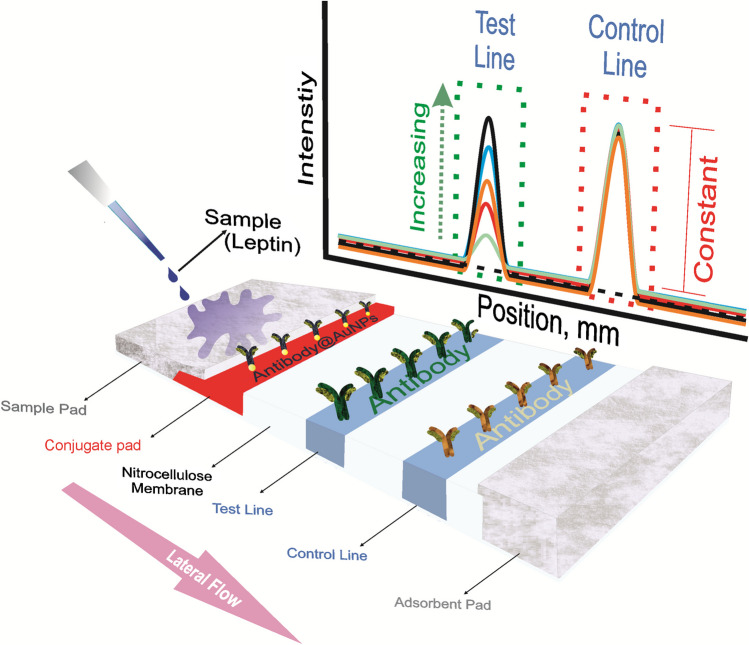
Schematic representation for working principle of LFA for leptin detection

In the assay shown in Fig. 1, the sample flows laterally through capillary action from the sample pad towards the conjugate pad and interacts specifically with the Ab@AuNPs (if leptin is present, antibody-antigen interaction) on the conjugate pad. It then continues to flow towards the adsorbent pad. Before reaching the adsorbent pad, the antibodies immobilized on the test line capture the solution, and the captured AuNPs (red-coloured) create a line pattern. Another portion of the flowing Ab@AuNPs solution, propelled by lateral flow, interacts with the anti-leptin selective antibody on the control line, forming a line pattern on this line as well. If the sample does not contain leptin, no interaction on the conjugate pad occurs, and the Ab@AuNPs pass through the test line. As a result, no pattern formation is observed on the test line, but the control pattern remains unchanged. In summary, the presence of two lines indicates a positive result, meaning leptin is present, while a single line indicates a negative result, meaning leptin is absent. In addition, the test line intensity increases with the increasing concentration of leptin without any change on the control line.

Furthermore, the detection limit, and stability were determined from the quantitative studies conducted at different leptin concentrations (0.5–500 ng/mL, Clinical standard: 2.5–15 ng/mL, [27]), using the colorimetric results. Within the scope of the study, the LFA developed for leptin was tested in human serum samples (commercial). A lateral flow reader device was employed for these quantitative studies. The general operating principle of this device is based on reading the increase in signal intensity of the test line, which is proportional to the amount of AuNPs that form visible lines in the sampled LFA placed in the sample chamber. An increase in this quantity is expected to be directly proportional to the analyte quantity.

## Results and discussion

### Characterization of AuNPs

**Fig. 2:**
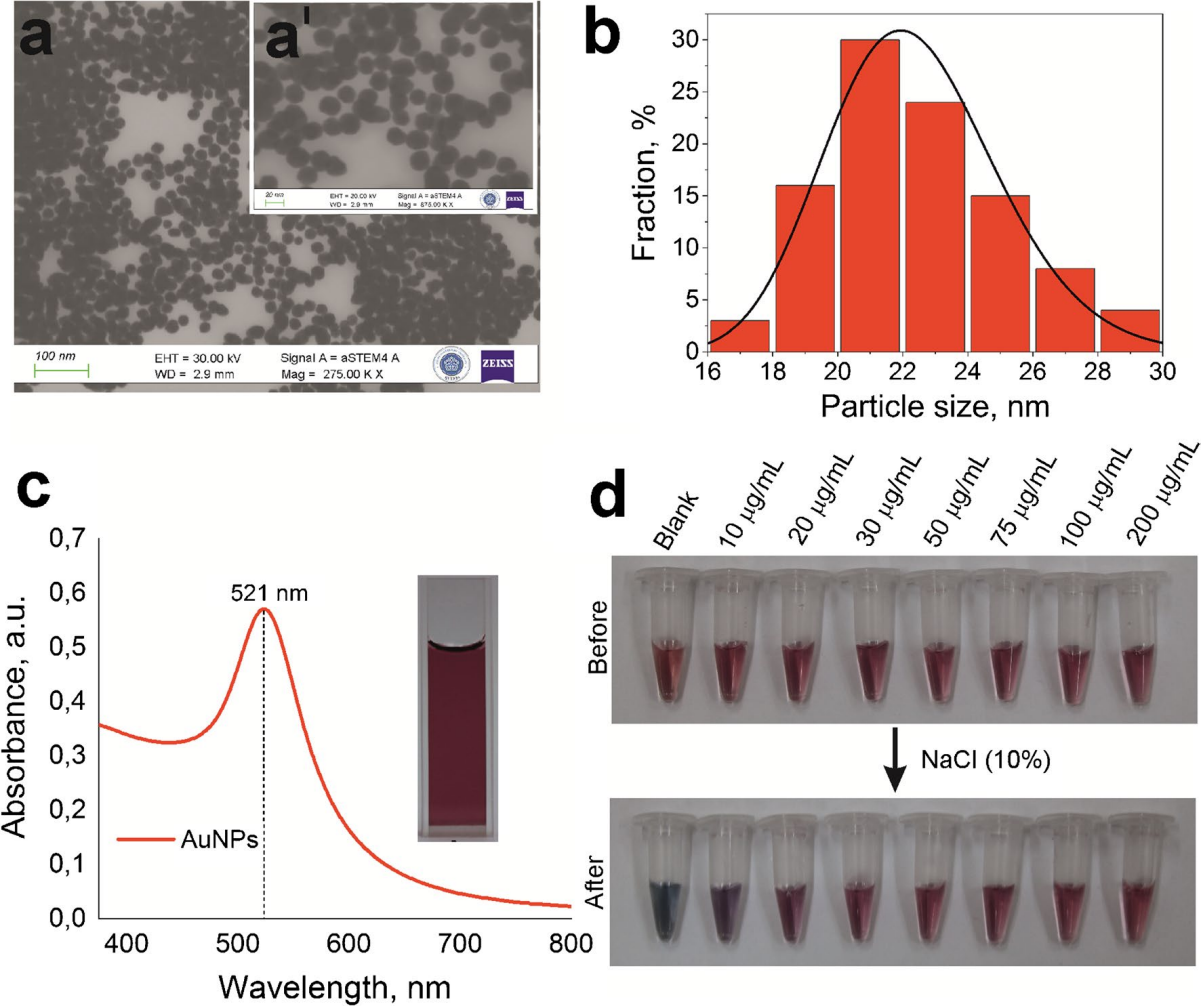
STEM images at different magnifications for AuNPs **(a- a')**, The size distribution analysis of AuNPs (**b**). The visible range of absorption spectrum and photo of AuNPs solution (**c**). Gold aggregation test: Images before and after the addition of 10% of NaCl solution to solutions containing different concentrations (0, 10, 20, 30, 50, 75, 100, 200 µg/mL) of anti-leptin antibody (**d**)

The morphological characterization of the obtained AuNPs were carried out using the Scanning Transmission Electron Microscopy (STEM) technique. STEM images of AuNPs at different magnifications are presented in Fig. 2a-a'. As seen herein, spherical particle structures were obtained. In addition to the morphological analysis results of AuNPs, a particle size distribution histogram was obtained by using the ImageJ program. In the graph given in Fig. 2b, the average particle size of the AuNPs was determined as 22.1 nm.

Figure 2c shows the visible range of absorption spectrum and photo of a AuNPs solution with a characteristic red colour. An absorption maximum was observed at 521 nm originating from the surface plasmon absorption of the dispersed AuNPs [27].

### Optimization of antibody quantity

In this step, the optimization of antibody quantity on AuNPs was carried out through aggregation test with a sodium chloride (NaCl) solution to find the optimal ratio for functionalizing the AuNPs surfaces with antibodies and to characterize if the AuNPs surfaces were entirely functionalized with antibodies [28, 29]. In this test, after the interaction of AuNPs with antibodies at various ratios (0–200 µg/mL), 20 µL of NaCl (10%) was added to the solution to monitor the aggregation of AuNPs. In case of aggregation, it indicated that the AuNPs' surfaces were not entirely covered with antibodies, and the addition of NaCl disrupted the charge balance, leading to aggregation. The lowest ratio without aggregation was considered the optimum amount and was used in the LFA design. For this purpose, the AuNPs solution was adjusted to pH 9.0 using a borate buffer (10 mM, pH 9.2) [12]. Then, different concentrations (0, 10, 20, 30, 50, 75, 100, 200 µg/mL) of anti-leptin antibody solutions were separately added to 150 µL of AuNPs solutions. These solutions were mixed at 25 ºC (600 rpm) for 30 min of incubation, followed by the addition of 20 µL of 10% NaCl solution. The resulting solutions were incubated under the same conditions for 15 min. As shown in Fig. 2d, when NaCl solution was added to the blank AuNPs solution, the colour changed from red to light blue, while it changed to purple in solutions containing 10 and 20 µg/mL of antibodies. It was observed that the colour of solutions containing 30–200 µg/mL of antibodies did not change, indicating that the nanoparticle surfaces were entirely covered at these concentration values. Although 30 µg/mL, 100 µg/mL, and 200 µg/mL antibody concentrations were suitable, a 50 µg/mL of antibody concentration was determined as the optimal antibody amount to ensure complete surface coverage for these concentration values and to use a smaller amount of antibodies to save antibody.

### Optimization of the test/control line width

**Fig. 3:**
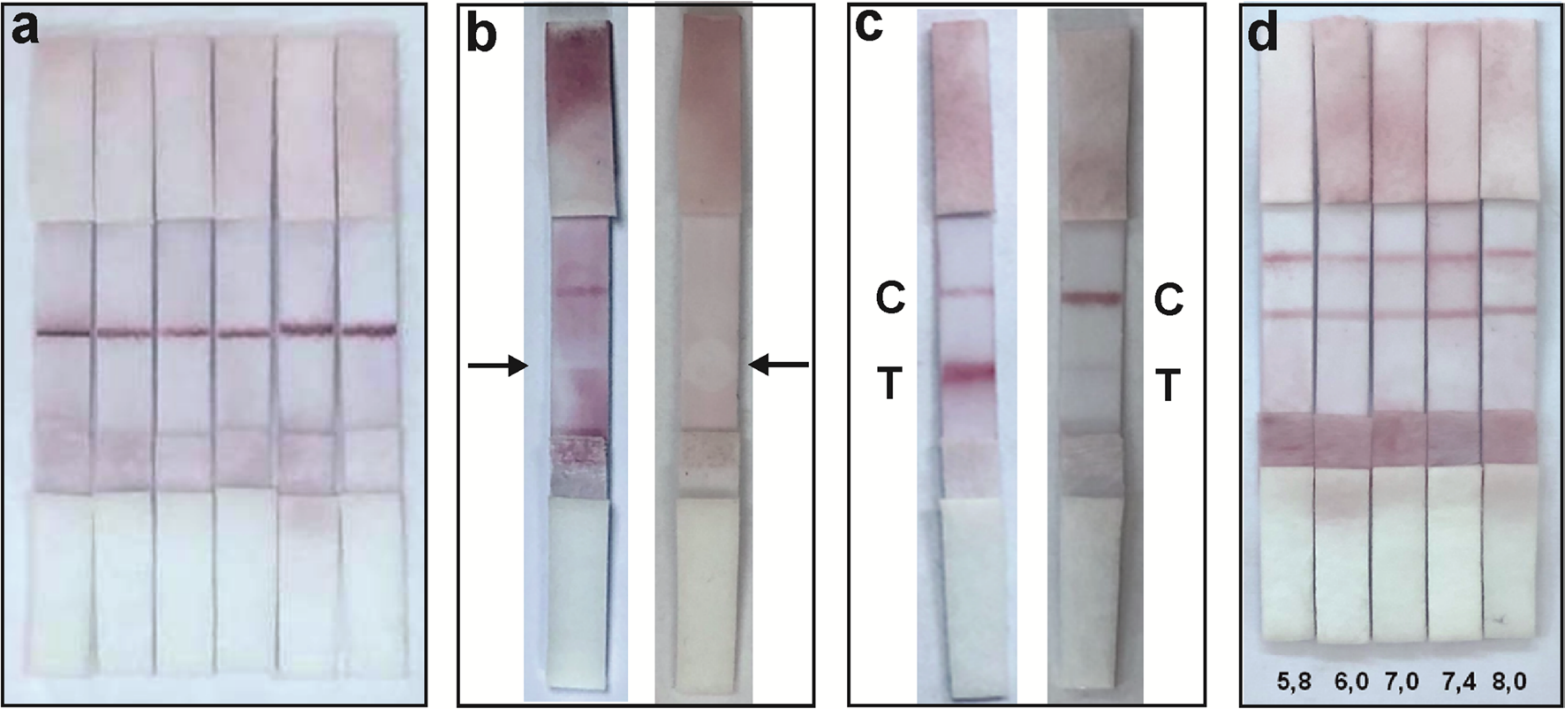
The image of the LFAs performed for the determination of leptin (500 ng/mL) with the detection pads prepared with different amounts of antibody spray (**a**). The image of the LFAs performed for the determination of leptin (500 ng/mL) with the detection pads prepared with excessive amounts of antibody spray (Stock-1 mg/mL) (**b**). The images of LFAs showing test and control lines obtained when the test antibody is printed thick and the control antibody is printed thin when the test antibody is printed thin and the control antibody is printed thick (**c**). The images showing test and control lines obtained for LFAs at different pH values (5.8; 6.0; 7.0; 7.4; 8.0 in PBS) of leptin solutions (**d**)

The experiments were conducted to ensure a clear visual observation of test results and to optimize the width of test/control lines and the amount of antibodies on the lines. In these experiments, the antibody concentration was varied between 10–200 µg/mL, and the test antibodies were deposited on the center of the membrane (Fig. S6). The studies on excess concentration of leptin determination (500 ng/mL) were conducted with the prepared LFAs. Figure 3a shows the images of LFAs to compare the antibody deposition and line width. As the line width and antibody amount increased, the width of the resulting red-coloured signal line also increased. An amount of 25 µg/mL with a line width of 1 mm was determined as the optimum experimental condition for the detection pad to achieve both a visible and homogeneous signal and to save the antibodies. This experiment also demonstrated the functionality of the test line antibodies since a signal was observed on the test lines, guiding the development of the qualitative/quantitative LFA for leptin in the further experimental step.

Figure 3b displays the images of LFAs conducted for leptin determination using membranes prepared with excess amount of antibody deposition (1 mg/mL). In this experiment, it was assumed that an excessive amount of antibody on the test line would result in a stronger signal. However, as seen in Fig. 3b, instead of the expected dark red line formed by the accumulation of nanoparticles on the test line with excessive antibody deposition, a white area formed. This effect was thought to be related to the detection pad, and LFAs were prepared by only dropping the test antibody stock solution onto the detection pad (resulting in a dot). When the target analyte leptin (500 ng/mL) was run, a similar white circular area formed as in the previous case. This interesting phenomenon has not been extensively discussed in the literature, however, it was seen that excessive antibody deposition may function as a surface-blocking agent, causing analyte-loaded nanoparticles to bypass this region and move along or within the membrane. In the further experiment, test and control lines were printed together and experiments were conducted. For this purpose, the antibody deposition was performed with one of the test and control lines being thick while the other was thin, and LFAs were prepared for leptin determination (500 ng/mL). As seen in Fig. 3c, when the test antibody was deposited thick, the test line created a thicker signal line, while the control line produced a weaker signal. Likewise, when the control antibody was deposited thick, the control line created a thicker signal line, while the test line produced a weaker signal. When both were deposited in equal amounts and with equal line thicknesses, the line thicknesses and signals were found to be equal (Fig. S7).

### Optimization of pH

In the studies conducted to optimize the operating pH, LFAs were prepared with the determined optimum preparation conditions. Leptin solutions were prepared at different pH values (5.8, 6.0, 7.0, 7.4, 8.0) with PBS and run on the prepared LFAs. Figure 3d shows LFAs conducted at different pH values, displaying the test and control lines obtained (see Fig. S7 showing only control line for blank PBS). As seen in the figure, both control and test lines were observed at all tested pH values, and it was observed that the lines obtained did not significantly change with pH. Therefore, a working pH value of 7.4 (as biological serum pH value) was chosen for subsequent experiments, as it provided suitable signals for commercial serum tests and based on the pH experiments conducted.

As a result of the conducted optimization studies above, it was determined that the optimum antibody concentration for conjugation was 50 µg/mL. Additionally, the optimum antibody concentration for the test and control lines was determined to be 25 µg/mL. The optimization of line width for test and control lines was carried out, and the most suitable line width was optimized at 1 mm. LFAs prepared with these antibody concentrations were observed to provide visible, high-intensity signals for leptin determination. In the studies conducted to determine the appropriate flow rate, it was observed that washing the conjugate pad with an immunobuffer solution facilitated the flow of AuNPs, and washing the sample pad and the detection pad changed the flow rate. Within the scope of the optimization studies, LFAs washed with an immunobuffer solution prepared with 3% BSA and 0.05% Tween-20 were found to have the most homogeneous flow and suitable flow rate. Additionally, experiments were conducted using a target analyte solution prepared with a pH = 7.4 phosphate buffer solution, resulting in a suitable signal.

### Determination of leptin with LFA

**Fig. 4:**
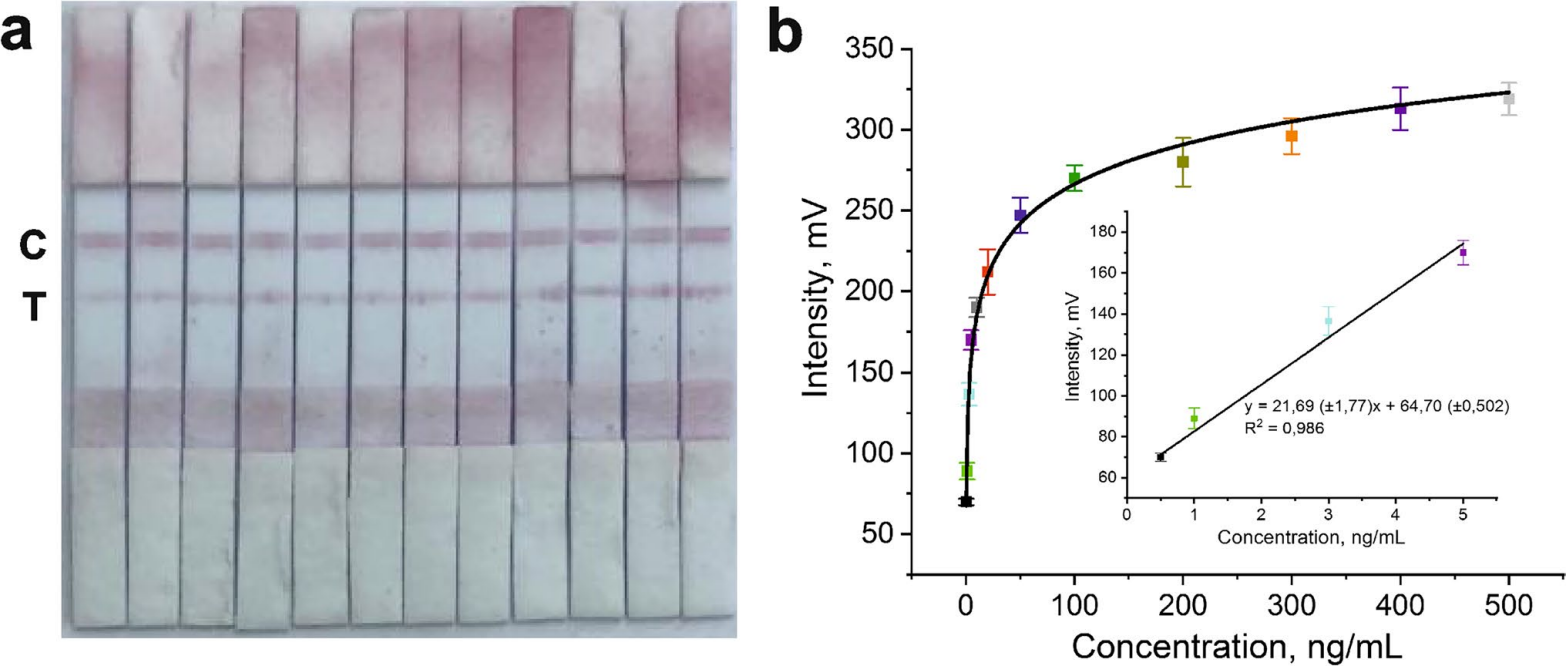
The images of LFAs conducted with a solution of commercial blood serum (pH = 7.4, PBS) prepared with different concentrations (from left to right; 0.5, 1, 3, 5, 10, 25, 50, 100, 200, 300, 400, 500 ng/mL) of leptin (**a**). The intensity values of signals *vs* increasing concentration of leptin (**b**). The inset shows the linear range curve for the intensity values of signals *vs* increasing concentration of leptin

The qualitative and quantitative determination of the leptin hormone was performed under optimum conditions using the prepared sensor. Initially, both the test and control lines were prepared with equal amounts of antibodies and equal line thicknesses in the LFAs, and qualitative determination of the excess amount of leptin (500 ng/mL) was performed. As seen in Fig. S7, when both lines were prepared with equal amounts and equal line thicknesses, it was observed that the line thicknesses and signal intensity were equal, indicating that the leptin hormone could be qualitatively determined. In the next stage, LFAs were prepared by printing antibodies for both the test and control lines with equal amounts and equal line thicknesses for semi-quantitative determination of leptin. Figure 4a shows the images of LFAs conducted with leptin hormone solutions prepared in commercial serum (tenfold diluted in PBS, pH = 7.4) at different concentrations (0.5, 1, 3, 5, 10, 25, 50, 100, 200, 300, 400, 500 ng/mL). The colour intensity of the test lines increased as the concentration of leptin hormone increased (from left to right), whereas the colour intensity of the control lines remained constant.

Furthermore, a lateral flow reader device was used to determine the intensity of the optical signals, which were used for the calculation of the limit of detection (LOD) and the limit of quantification (LOQ) from the colorimetric results obtained through quantitative studies conducted at different leptin concentrations. The general working principle of the lateral flow reader is based on reading the increase in signal intensity of the test line depending on the amount of AuNPs that form the lines visible in the sampled LFAs placed in the sample chamber. Figure 4b illustrates the changes in signal intensity with concentration when LFAs conducted with leptin hormone solution prepared in commercial serum (tenfold diluted in PBS pH = 7.4) at different concentrations (0.5–500 ng/mL) were analyzed. Within the region showing a linear increase in the range of concentrations from 0.5 to 5 ng/mL (R^2^ = 0.986), as shown in the inset of Fig. 4b. The LOD value was calculated as 0.158 ng/mL, and the LOQ value was calculated as 0.479 ng/mL, using LOD = 3.3σ/S and LOQ = 10σ/S, where σ is the standard deviation of the blank signal and S is the slope of the regression line [30].

### Performance in real samples: Stability, spike, and recovery

Stability for a developed method is a measure of its durability within a specific environment, under the intended storage conditions, or under the influence of other environmental factors [31]. Both short- and long-term stability studies were conducted for the LFA system developed for leptin determination. For this purpose, LFAs were prepared and stored in aluminium foil at room temperature. The measurements were conducted with a 3 ng/mL of leptin solution. Fig. S8 shows the response of LFAs in short-term stability studies after 2 and 24 h, and in long-term stability studies after 1 month and 6 months.

The results calculated according to the calibration curve, standard deviations, and relative standard deviations (RSD %) are presented in Table 1. According to the results obtained from short-term stability studies, no significant change in the test response of the LFA occurred within the first 24 h of the tests. The results for the long-term stability studies showed that although a slight decrease in the test response was observed over 6 months, the responses are very close to the added amount. The reason for this decrease may be attributed to the fact that, despite the LFAs being stored in aluminium foil, they did not undergo a packaging process like that of commercial products, and thus, there might have been slight exposure to air. Furthermore, RSD values calculated for the accuracy of the developed method falls within the acceptable range of 10–15% for concentration levels in biological samples [31].

**Table 1:** Short- and long-term stability results for the developed LFA for leptin hormone determination

Short-term stability (*n *= 3)					Long-term stability (*n* = 3)				
**Added (ng/mL)**	**Found (ng/mL)**				**Added (ng/mL)**	**Found (ng/mL)**			
	**2 h**	**RSD%**	**24 h**	**RSD%**		**1 m**	**%RSD**	**6 m**	**RSD%**
3.0	3.3 ± 0.3	9.1	3.3 ± 0.3	9.1	3.0	2.9 ± 0.3	10.3	2.7 ± 0.4	14.8

In the scope of the study, to determine the accuracy of the developed LFA test results, a commercially approved method was used for comparison. For this purpose, ELISA tests, which are one of the gold standard methods, were performed using four different concentration values within the determined working range (*n* = 3). Since the working range of the ELISA test kit was 15.63 pg/mL to 1000 pg/mL, the samples used in the LFA test were diluted 10 times before analysis. The analysis results obtained were multiplied by 10 and converted to ng/mL. Table 2 shows the results of leptin analysis in artificial serum samples conducted using the developed LFA and the reference method ELISA. While the recovery values obtained with ELISA for the given concentration values ranged from 90 to 100%, they were calculated to be between 95 and 110% in the developed method. The obtained recovery values demonstrate that the developed LFA can be used for leptin [31].

**Table 2:** Leptin analysis in artificial serum samples using LFA and ELISA methods

Leptin LFA				Leptin ELISA		
Sample	**Added (ng/mL)**	**Found (ng/mL)**	**Recovery (%)**	**Added (ng/mL)**	**Found (ng/mL)**	**Recovery (%)**
1	1.0	1.1 ± 0.1	110	1.00	0.9 ± 0.1	90
2	2.0	1.9 ± 0.1	95	2.00	2.0 ± 0.2	100
3	3.0	3.3 ± 0.3	110	3.00	2.9 ± 0.3	97
4	4.0	3.8 ± 0.3	95	4.00	3.6 ± 0.3	90

## Conclusion

In summary, an LFA for the detection of leptin was designed and tested, being capable to achieve the detection at ng/mL levels in commercially obtained real samples. As a result of the quantitative analysis, the LOD value was calculated as 0.158 ng/mL and LOQ was calculated as 0.479 ng/mL for leptin hormone. Short- and long-term stability studies of the developed LFA showed that the test response was equal even after 6 months. Compared with the ELISA method, the LFA accuracy tests performed on commercial and artificial serum samples, the recovery values obtained between 95 and 110% show that the developed LFA can be used as a novel PoC diagnostic technology for quantitative analysis of leptin hormone. The concentration range of leptin hormone in actual clinical samples is 2.5–15 ng/mL. In this study, the experiments were performed between 0.5 and 500 ng/mL of leptin hormone. It is important to note that even in the presence of 25 and 50 ng/mL, there is an increase in the intensity of the signal however the sensitivity decreases at these quantities of analyte. Therefore, as a drawback of the proposed LFA, the developed assay is more useful for qualitative detection of leptin hormone for the high concentration values (above 5 ng/mL).

## Supplementary Information

Below is the link to the electronic supplementary material.

Supplementary file1 (DOCX 2009 KB)

## Data Availability

No datasets were generated or analysed during the current study.
