# Supplementary material for: Quantitative determination of leptin hormone using gold nanoparticle-based lateral flow assay
Source: Mikrochim Acta. 2025 Jan 9;192(2):63. doi: 10.1007/s00604-024-06945-6 (PMC11717784; doi:10.1007/s00604-024-06945-6)
Supplement: Supplementary file 1 — Supplementary file1 (DOCX 2009 KB) [file 604_2024_6945_MOESM1_ESM.docx]

**Supporting Information**

**Quantitative analysis of leptin hormone using gold nanoparticle-based lateral flow assay**

Erhan Zor^1,2*^, Sabri Alpaydin^3^, Haluk Bingol^1,4^

*^1^Science and Technology Research and Application Center(BITAM), Necmettin Erbakan University, Konya, Turkey*

*^2^Department of Science Education, A.K. Education Faculty, Necmettin Erbakan University, Konya, Turkey ^3^Department of Chemistry Education, A.K. Education Faculty, Necmettin Erbakan University, Konya, Turkey*

*^4^Department of Basic Sciences, Faculty of Engineering, Necmettin Erbakan University, Konya, Turkey*

***Corresponding Author:**

Erhan Zor (Assoc.Prof. Dr.); zorerhan@gmail.com; ezor@erbakan.edu.tr

**1. Preparation of antibody conjugated AuNPs**

The synthesis of colloidal AuNPs was carried out according to the Turkevich method [1]. To this aim, all glassware and magnetic stirrer bars used in the synthesis procedure were cleaned with aqua regia (HNO_3_/HCl=1:3, v/v), rinsed with pure water, and then dried in the oven before use. A solution of 0.5 mL of HAuCl_4_ (12.5 mM) in 50 mL of H_2_O (Milli-Q) was boiled under vigorous stirring. Then, typically, a sodium citrate solution (1%, 1.25 mL) was rapidly added into the boiled HAuCl_4_ solution and stirred for 10 min while observing the characteristic colour change from transparent to deep blue and then to wine-red (Fig. S1). After cooling at ambient temperature with stirring, the resultant solution was wrapped with aluminium foil to protect it from light and stored at +4 ºC.


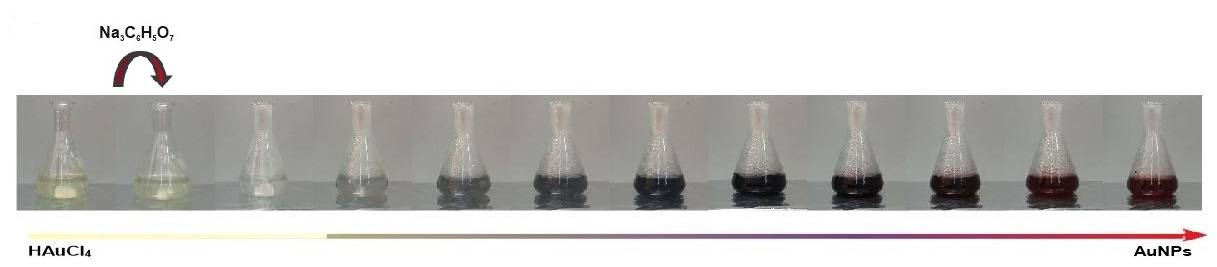


Figure S1. The synthesis procedure of gold nanoparticles (AuNPs).

The surface modification of colloidal AuNPs obtained in the previous step was performed. It has been reported in the literature that this process is achieved through strong thiol bonds that can be formed between AuNPs and the sulphur atom present in the cysteine groups of antibodies, and electrostatic interactions by mixing appropriate proportions of AuNPs and antibody solutions [2–4]. In the functionalization process, a mixture of AuNPs (adjusted to pH 9 using 10 mM borate buffer) and antibody (adjusted to the desired final concentration using antibody stock solution; except aggregation tests, adjusted to 50 µg/mL) was prepared in a beaker (Fig. S2a-b) and transferred into Eppendorf tubes. It was then mixed for 20 minutes at 650 rpm using a cooled shaker and transferred into the tubes (Fig. S2c-d). After this process, 1% BSA was added to the solution and mixed again at 650 rpm for 20 minutes at +4 ºC (Fig. S2e-g). Subsequently, the resulting mixtures were subjected to a centrifugation process at 20000 rcf for 20 minutes to precipitate the conjugated antibodies and functionalized AuNPs (Ab@AuNPs) (Fig. S2h-i). The pellet was separated from the supernatant (Fig. S2j) and then re-dispersed in borate buffer (2 mM, pH 7.4, + 10% sucrose) using vortex mixing for further use (Fig. S2k-l) [5]. The successfully obtained antibody-conjugated AuNPs (Ab@AuNPs) were used in the preparation of the conjugation pad, guided by the optimization studies conducted in Section 3.2.

**
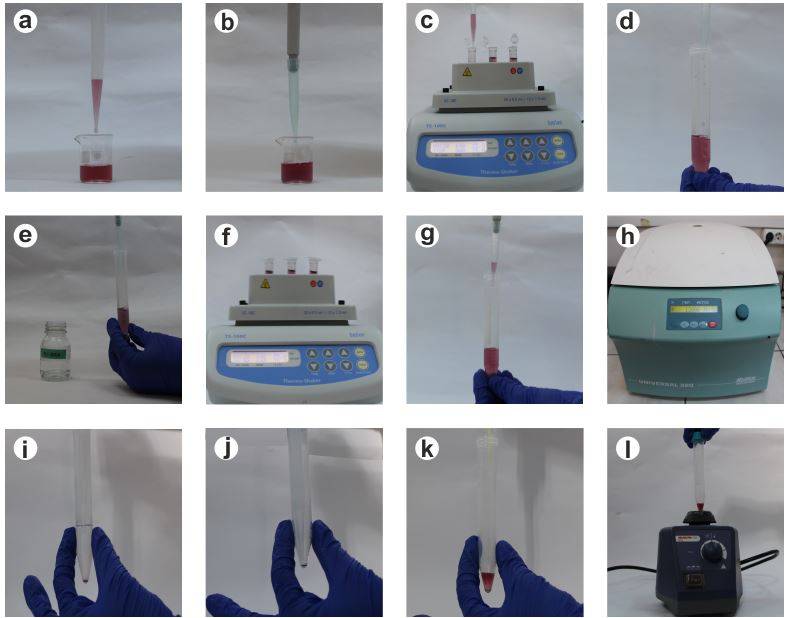
**

**Figure S2.** The experimental demonstration of surface functionalization of AuNPs with antibody. Mixing antibodies with AuNPs **(a-b)**. Mixing the solution taken into Eppendorf tubes with the help of a refrigerated shaker at 650 rpm for 20 minutes at 37 ºC and transferring it to the tube **(c-d)**. Add 1% BSA to the solution and mix again at 650 rpm for 20 minutes at +4 ºC **(e-f-g)**. Precipitation of the resulting mixtures at 20000 rcf **(h-i)**. Separation of precipitated antibody-functionalized AuNPs (Antibody@AuNPs) from the supernatant solution **(j)**. Re-disperse by vortexing in borate buffer (2 mM pH 7.4 + 10% sucrose) **(k-l)**.


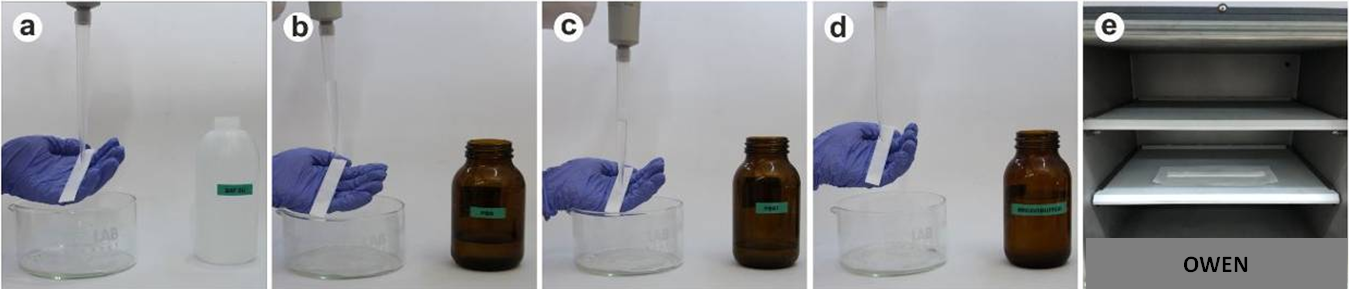


Figure S3. The photographs of washing the adsorbent and sample pads (a-d), and drying in an owen (e).


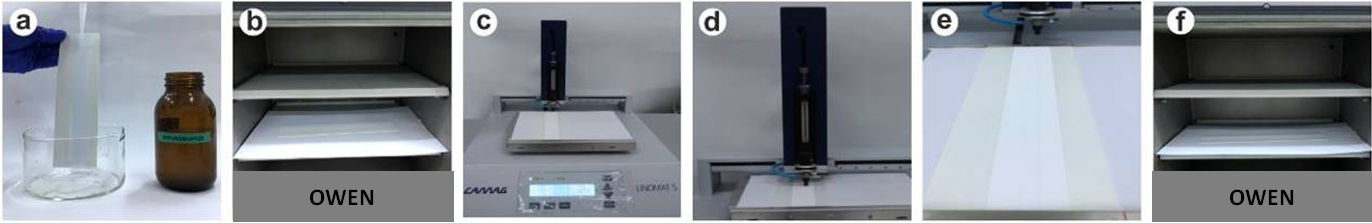


Figure S4. The photographs of washing the test membrane (a), drying in an owen (b),

and spraying test/control lines (c-e) and drying in an owen (f).


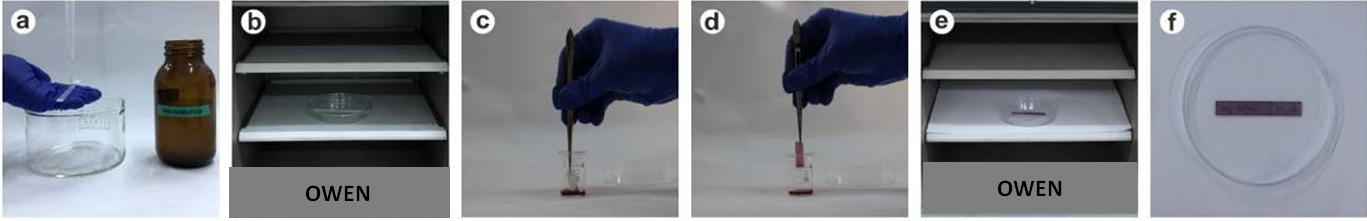


Figure S5. The photographs of washing the conjugation pad (glass-fiber) (a) drying in an owen (b), and dipping in AuNPs solution (c,d) and drying in an owen (e). The photograph of the dried conjugation pad (f).

**2. Optimization of LFA for leptin detection**

**2.1. Optimization of washing process for pads**

In this optimization stage, the tests were conducted with the prepared LFAs to optimize the lateral flow of the sample and AuNPs in the conjugation pad on the created platform. For this purpose, the impact of washing and non-washing conditions of the conjugation pad and the test membrane on the sample flow was examined. At this stage, no treatment was applied to the conjugation pad (glass fiber). For optimum lateral flow, the movement of AuNPs in the conjugation pad of the sample was monitored. The experiment results indicate that when no treatment was applied to the conjugation pad, nanoparticles did not flow through the LFA membrane. It was observed that washing with immunobuffer caused the solution to be retained on the test membrane and flow more slowly.

The same procedures were also carried out with LFAs by washing with immunobuffer before preparing the conjugation pad. The results indicate that the washing process of the conjugation pad facilitates the flow of AuNPs on the test membrane. Furthermore, it was observed that washing the pads and test membranes also affected the flow. In LFAs, the flow rate is crucial for the interaction of the analyte in the sample with the Ab@AuNPs in the conjugation pad and for the interaction with the antibodies added to the test and control lines during flow. Very fast flows can lead to inadequate interactions, while very slow flows may prevent the sample from reaching the adsorbent pad and cause drying on the test membrane [5]. Therefore, in further experiments, different amounts of BSA and Tween-20 were examined to control the flow rate and prevent non-specific surface interactions with lateral flow. The studies were conducted to determine the optimal conditions by preparing immunobuffer solutions with Tween-20 concentrations ranging from 0.01% to 0.1% (0.01, 0.025, 0.05, 0.075, 0.1%) and BSA concentrations between 0.5% and 5% (0.5, 1.0, 3.0, 5.0%). For this purpose, the experiments were first conducted with the membranes washed with a solution containing 0.5% BSA and varying amounts of Tween-20. The experimental results indicated that when both BSA and Tween-20 concentrations were low, the AuNPs did not flow on the LFA, and only the aqueous solution flowed. As the Tween-20 concentration increased, it was observed that both the flow rate increased and AuNPs exhibited a homogeneous flow on the membrane. In the further experiments, the use of a solution containing 1% BSA and varying amounts of Tween-20 indicated that when Tween-20 concentration was low, the nanoparticles exhibited intermittent, non-continuous flow on the LFA, and only the aqueous solution flowed on the membrane. As the Tween-20 concentration increased, it was observed that the flow rate increased and the nanoparticles exhibited homogeneous flow. Furthermore, when compared to a 0.5% BSA concentration, it was observed that the flow rate slowed down. The use of a solution containing 3% BSA and varying amounts of Tween-20 showed that the nanoparticles did not flow, and only the running solution flowed on the LFA as Tween-20 concentration was low. As the Tween-20 concentration increased, it was observed that both the flow rate increased and the nanoparticles exhibited homogeneous flow. An interesting ratio to note is 3% BSA and 0.05% Tween-20, where AuNPs exhibited homogeneous flow and demonstrated an average flow rate. In the last experiments, the use of a solution containing 5% BSA and varying amounts of Tween-20 indicated that when both BSA and Tween-20 concentrations were high, the nanoparticles did not flow on the LFA, and only the solution flowed. An interesting ratio to note is 5% BSA and 0.01% Tween-20, where the nanoparticles exhibited homogeneous flow and demonstrated an average flow rate at the beginning. However, it was observed that the nanoparticles did not flow after a certain time and dried on the test membrane in the experiments of LFAs prepared with these ratios.


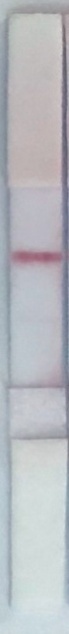

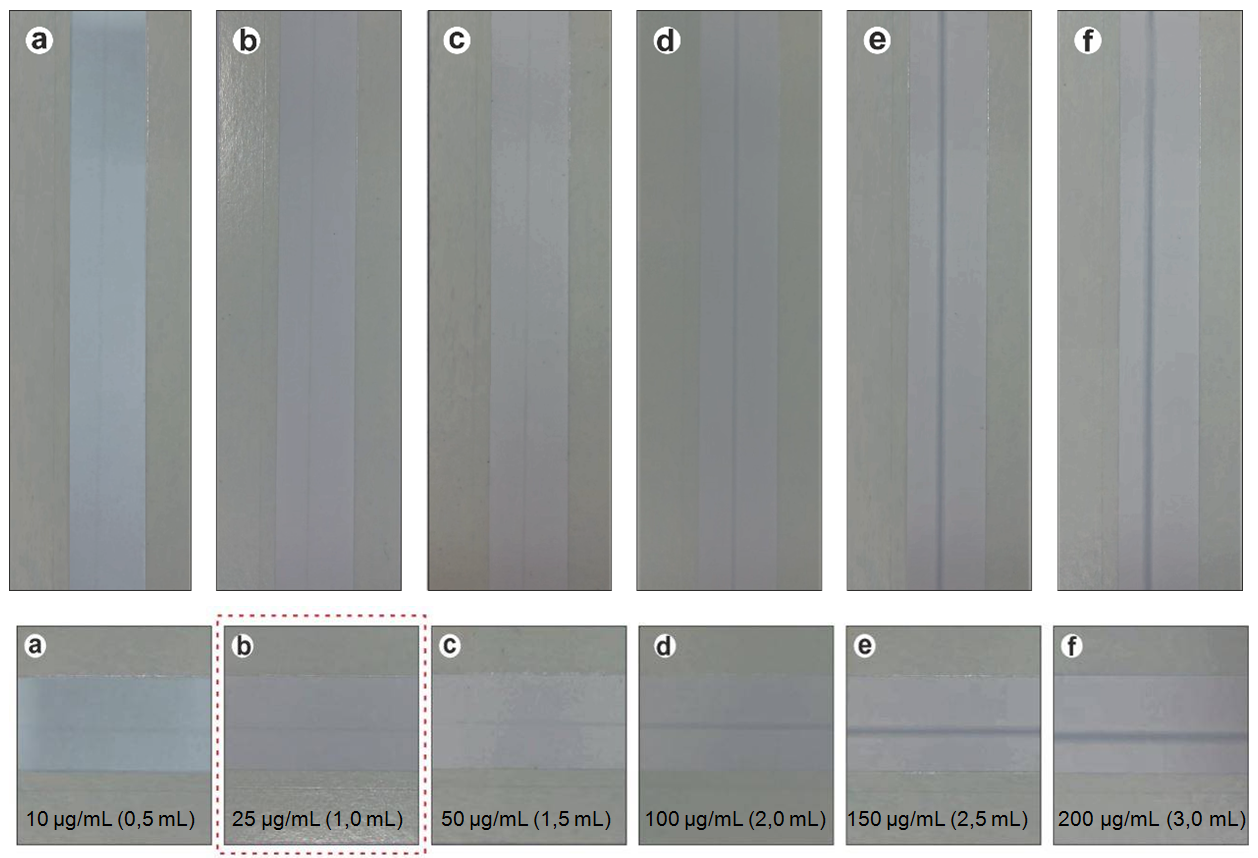


Figure S6. The photographs taken on the full-size membrane and of the membranes on which the antibody was sprayed, placed side by side to compare line widths (a-f).

**T C**


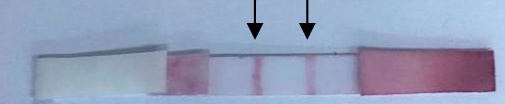


Figure S7. The photograph showing the test line in PBS (blank) and test/control lines obtained for LFA, in which antibody lines were sprayed in equal amounts and with equal line thicknesses (The concentration of leptin is 500 ng/mL).

**
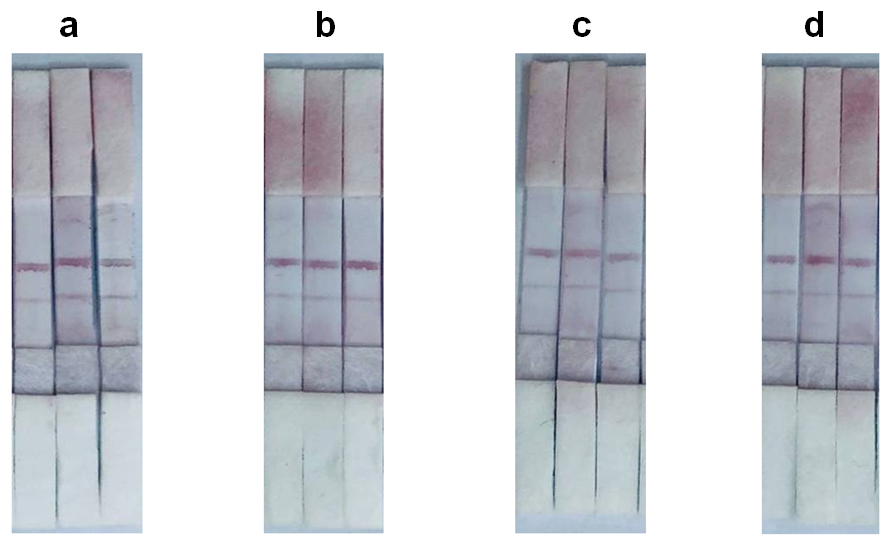
**

Figure S8. The photographs of the results obtained for LFAs for short-term (2 hours (a) and 24 hours (b)) and long-term (1 month (c) and 6 months (d)) stability experiments at a concentration value of 3 ng/mL.


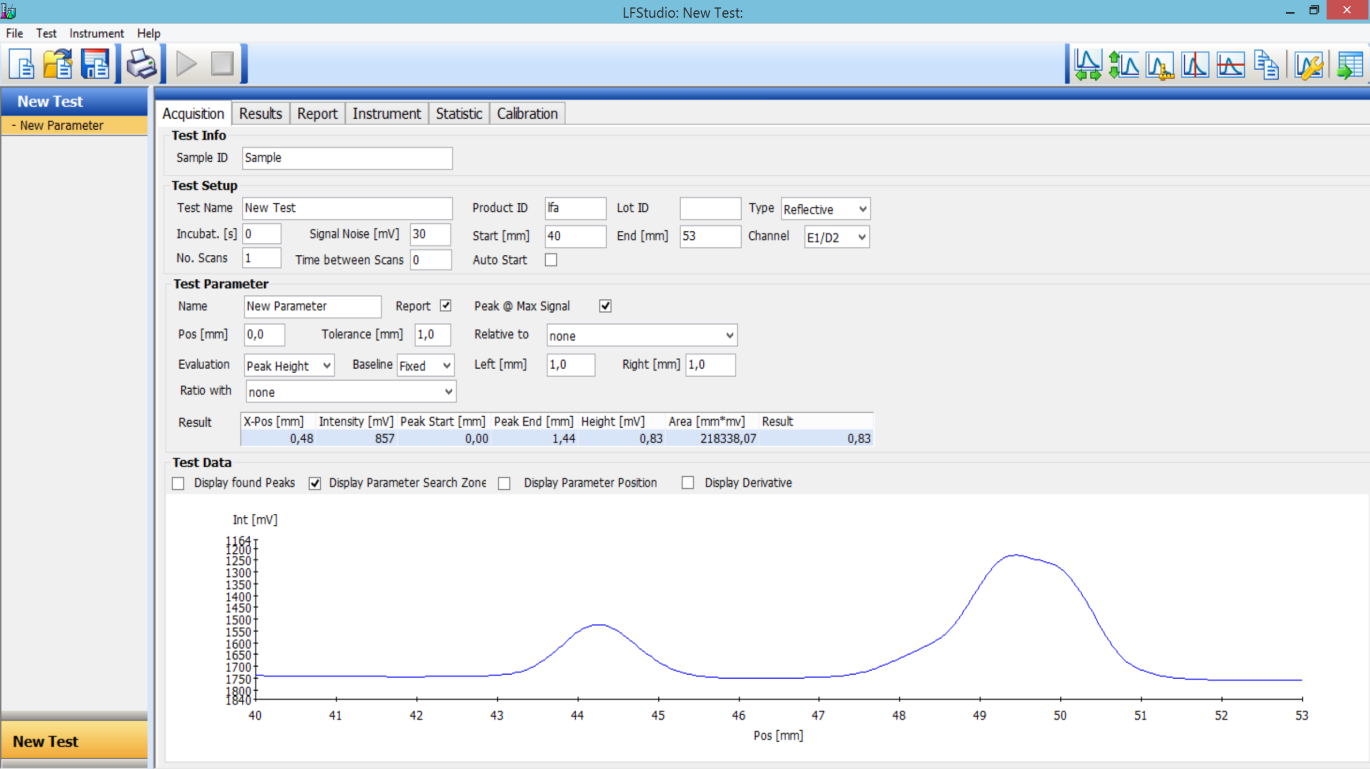


**Figure S9.** The screenshot showing the image acquisition on the lateral flow studio software.

**References**

[1] J. Turkevich, P.C. Stevenson, J. Hillier, A study of the nucleation and growth processes in the synthesis of colloidal gold, Discuss. Faraday Soc. 11 (1951) 55–75. doi:10.1039/DF9511100055.

[2] D. Quesada-González, C. Stefani, I. González, A. de la Escosura-Muñiz, N. Domingo, P. Mutjé, A. Merkoçi, Signal enhancement on gold nanoparticle-based lateral flow tests using cellulose nanofibers, Biosens. Bioelectron. 141 (2019) 111407. doi:https://doi.org/10.1016/j.bios.2019.111407.

[3] D. Quesada-González, G.A. Jairo, R.C. Blake, D.A. Blake, A. Merkoçi, Uranium (VI) detection in groundwater using a gold nanoparticle/paper-based lateral flow device, Sci. Rep. 8 (2018) 16157. doi:10.1038/s41598-018-34610-5.

[4] A. Chamorro-Garcia, A. de la Escosura-Muñiz, M. Espinoza-Castañeda, C.J. Rodriguez-Hernandez, C. de Torres, A. Merkoçi, Detection of parathyroid hormone-like hormone in cancer cell cultures by gold nanoparticle-based lateral flow immunoassays, Nanomedicine Nanotechnology, Biol. Med. 12 (2016) 53–61. doi:https://doi.org/10.1016/j.nano.2015.09.012.

[5] C. Parolo, A. Sena-Torralba, J.F. Bergua, E. Calucho, C. Fuentes-Chust, L. Hu, L. Rivas, R. Álvarez-Diduk, E.P. Nguyen, S. Cinti, D. Quesada-González, A. Merkoçi, Tutorial: design and fabrication of nanoparticle-based lateral-flow immunoassays, Nat. Protoc. 15 (2020) 3788–3816. doi:10.1038/s41596-020-0357-x.
